# Interactions Between Lamotrigine and Contraceptives – Communication Practises

**DOI:** 10.1192/bjo.2024.607

**Published:** 2024-08-01

**Authors:** Samina Monir, Cissy Atwine, Helen Hutchings, Michael Harris

**Affiliations:** Sussex Partnership NHS Foundation Trust, Worthing, United Kingdom

## Abstract

**Aims:**

This audit assesses communication practices regarding interactions between lamotrigine and oral contraceptives in North West Sussex (NWS) Specialist Perinatal Mental Health Services (SPMHS).

The predicted outcome includes increasing awareness about potential interaction between lamotrigine and contraceptives with resulting impact on patient safety.

**Background:**

Lamotrigine is used for epilepsy and mental health disorders but can interact with contraceptives, affecting efficacy and safety. NICE recommends it for bipolar depression, relapse prevention and recurrent depression. Interactions with hormonal contraceptives can influence effectiveness of either drug and increase the risk of side effects. Patients on lamotrigine should be counselled so they can make an informed decision about taking the medication.

**Methods:**

Reviewed records of all patient on the caseload on 21^st^ June 2023. Collected data for lamotrigine prescription, indication, contraceptive method, and documented counselling. Calculated percentage of patients counselled on lamotrigine-contraceptive interaction.

**Results:**

In 261 patient records, 11.9% were previously or currently on lamotrigine or had a discussion about starting lamotrigine. 6.1% currently and 3.1% previously on lamotrigine. Counselling on lamotrigine's interaction with oral contraceptives was documented with 3.1%, while 74.2% received none. Indications for lamotrigine use were epilepsy 9.7% and mood stabiliser 90.3%. Of 27 patients who weren't currently pregnant, 9 of them were informed of the interaction risk while 18 were not. Contraception methods were documented for 10 individuals.

**Conclusion:**

Findings showed the need for increased awareness about the interaction and documentation of appropriate discussions to inform their choice.

